# New Jersey State Dental Society

**Published:** 1889-01

**Authors:** 


### NEW JERSEY STATE DENTAL SOCIETY.

JULY, 1883.

ESPECIALLY REPORTED FOR THIS JOURNAL.

### Discussion on Dr. Codman's paper, Enforced Climate and Diet as Affecting the Teeth.

*President Brown*—I want to thank Dr. Codman, not only for the society, but personally. The paper he has read gives a clincher to the one which I had the honor of reading before this society last year; and I hope that Dr. Atkinson, who was to have opened the discussion of my paper, will open the discussion of this one.

*Dr. Atkinson*—It is difficult to add anything to the trend of the research and of prophecy that we have been listening to. I have nothing but commendations for the general principles of the paper and for the statements made; and the only criticism I have to offer is that the verbiage does not always fit. A musular brain is that which is not known to my anatomy; and I would suggest that muscular be changed for robust, or something that would more nearly comport with the magnificence of the portrayal and the beautiful correlation of aphorisms that indicate a research very uncommon.

That we all do live upon something that he has referred to, but not named, is generally accepted, I think, by all thinkers; and we are near the time when such deep philosophical disquisitions shall not only be tolerated, but received with delight and in some measure comprehended.

But all that is swallowed up in the immediate question, what shall be the next step; and the fact of each individual having to take the step that shall best comport with his particular environment, and that these communities that have been referred to may be sufficiently alike to walk together in obedience to the law that governs the food supply, and the preparation of the food when it has been attained, will enable us to come a little nearer to what is now engaging the minds of the so-called micologists, those who are studying the earliest embodiment of this power that has been so difficult to define, and the understanding of which is absolutely necessary to the comprehension of the question of food and nutrition. A late writer says that the function of the white blood corpuscles, the leucocyte in the mammalian circulation, is to eat the microbes that are inimical to the organization, and thus bring about a tolerable condition so that the other modes of the stored radiancy may awaken the primal steps in the process of building and nourishing the organs of the body.

When, some years ago, a few inspirations were spoken of by your humble servant before a body of this kind they would say, "Come out of the cloud; talk practical sense; we do not want any of these vagaries;" but before they would be through they would refer to the very same relations that we are trying to unravel in order to understand what our duty is.

There was something said about honesty and a commercial age. Commercialism is right if you use it right; and so is every other condition if we only know how to meet it. But how do we know how to meet it if we do not agree in our understanding and interpretations of things by referring back to the power that produces them?

As to the earth being once clothed with ice, it is altogether unphilosophical and contrary to true evolution, the stages of which do show us a great many things that have been attributed to the Devil's Mountain, which, if we had such a mountain, we might have sympathy with him, and work as brothers together in our investigations, and know that we know so little that we ought to be ashamed to say that we have grasped the problem of organized beings and settled the question of embodiment of soul; because in every human soul there has been planted both intellect and affection; there is a sense of brotherhood and of righteousness; a sense of doing to others as you would be done by; and that is not what has been called in this paper physics. I wish to God the word physics could be expunged from our nomenclature. There is no physics without that which produces natural organisms. There is no organ without that which gives the lay-out for the organ. There is the spirit within it—though it escapes us when we are not able to do anything more than mentally grasp at it—which is the doing power that reveals the truth to us.

I hope this paper will be discussed by the society, and all done that can be done to engage the stored radiancy that is somewhat obscured in it, so that we may be illuminated by an understanding of the laws that govern individual existences that have in them the intellect and affection that raises this question of morality.

*Dr. Thayer*—The important question raised in the paper seems to be: What shall a man eat thereby to have good mental and physical organization? I cannot give any better answer than that which is recorded in the Bible; where it speaks of that land which the people of Israel were going to occupy as filled with corn and wine and oil, and flowing with milk and honey. If these are the things which were good for men to enjoy in those days, why not take the old Bible and settle the matter?

*Dr. Stockton*—Dr. Codman spoke in his paper of the crude diet of the old New Englanders; how they lived on the plain foods of that time; and we know that they were mentally a grand race, we know what has sprung from them, but we do not know much about their teeth. I would like very much to learn something about them from the doctor. I consider this an important and practical question, one that brings us down out of the clouds to the comparative results and desirability of living on the plain, coarse foods used at that time, and the diet which we should have to-day. I doubt very much whether the race of to-day is mentally any stronger than they were in those old New England days. They were grand men.

*Dr. James Truman*—Mr. President, I was very much interested in the remarks of Dr. Codman, and I was glad that he brushed away all the old ideas that have been handed down through the books, and based his remarks entirely upon scientific research. He gave the opinion that food has very little to do with the character of the teeth as we find them. When I examine the teeth of those who live in the cold regions, as the Esquimaux, who live entirely upon a fatty diet, I find them of good character and structure; and as I pass to other regions, where the people live entirely upon a vegetable diet or a fruit diet, I find their teeth of the same general character as those of the meat and fat eaters. Also, when we examine the teeth of the early races of this continent, or in any quarter of the globe, we find the same character of teeth associated with the greatest variety of food habit. The question arises whether a meat diet or a vegetable diet is best for the human race. I hold that we may eat almost any form of food, but that we are not built for a meat diet. This is my positive conviction. I know that it is contrary to general opinion, and the shape of the teeth, especially of the canines, is cited in opposition to it. It is a fact that the human animal has comparatively smaller teeth than many of the herbiverous animals; that does not indicate a meat diet. We now live upon a mixed diet, but I think the time will come when the human race will refuse to eat anything that "has just died," as Dr. Atkinson puts it. To my mind the whole human race is gradually advancing from a coarse stage of existence, from a time when the flora and fauna on the earth's surface were rude and coarse. Yet that is an expression which we cannot properly use in this connection, because there is nothing coarse or fine in nature; but still there is a difference in the grain, not only in organized beings, but in vegetables that then existed; they were coarser in grain and coarser of organization than those of the present, and probably those of the future; and the reason, I think, lies in the fact that the food of those animals and vegetables was in a comparatively coarse and crude condition. The earth has been going through a refining process, as it were, and we find a different race of animals and a different race of men; and this change will go on until that condition which Dr. Codman has spoken of will come, when the earth will be peopled by a race of men and women who will not only not eat meat, but will probably be so spiritually improved by the better food taken into their systems that they will be an entirely different race from that which now exists. I believe that all nature points to that result. I believe that not only this earth, but the great series of worlds, every one of which is, or will be, peopled similarly to ours, must eternally advance toward the good in the refinement and elevation of the races which exist upon them.

*Dr. R. B. Winder*—MR. PRESIDENT AND GENTLEMEN:—I can speak in commendation of this paper, as I should in regard to everything that involves earnest scientific research. That our race will eventually feed only upon grain and fruits I am very much disposed to doubt. That food stuffs have an influence, both upon plant and animal life, is a well-established fact in physiology. We all know the decided influence and effect of coloring matter in the food stuffs of animals. Whether meat or vegetable food stuffs is to be the food of man in the future is a question which the future alone can decide. That we are not at present intended to be strictly graminivorous, we have abundant evidence, I think, in the anatomy of man. All those animals which live exclusively upon a grain diet have a series of stomachs. Man has but one; and every animal with a digestive apparatus that is very similar to ours is omnivorous in its food habit, so far as I have gone in biological research. So, reasoning from analogy and from the anatomy of man, I could not arrive at any such conclusion as that the food of the race will eventually be grain and fruits only.

The question which Dr. Stockton asked Dr. Codman is a very pertinent one, and I would like to have it answered, because I have been told by much older gentlemen than Dr. Codman that there has been a change in the structure of New England teeth. I have been told that, over and over again, by our old and honored brother, Dr. Riggs, of Hartford, Conn. But to say that our food stuffs control us is to go into a discussion of the chemical assimilation of food stuffs. The same food stuffs produce in different animals very different epidermic structure. The teeth belong to the epidermic structure, so that, while food has an influence, it does not control. We know that in the half-breed Indian, the child of an Indian mother and a white man, when there has been no change of climate nor of habits, and also no alteration in food supplies, there is a most material difference in the structure of the teeth. There we have to combat, the moment the chain is broken, that very troublesome thing, heredity, of which we know so little. I have given considerable thought during the past fifteen years to this subject, and there seems to be a debateable ground, where the facts rise and rebut each other, leaving you in a labarynth of mystery in regard to the matter, which, up to this time, no man has unraveled. There is not enough known about it to make much impression upon the general public. If we reason from analogy, from the food stuff which the infant takes, we would conclude that our diet should be animal as well as vegetable. If we are to draw any imaginary deduction from the character of the food which nature first givest to the child, we must inevitably come to the conclusion that man is not to be sustained without a certain amount of animal food. But take any avenue, and pursue it as you please in this direction, and you find rebutting testimony, and you are bewildered.

*Dr. Bailey.*—I have been very much interested in the discussion, and although it is a subject to which I have not given as much study as some others have, yet I have considered it somewhat. Dr. Winder has expressed very fully my thought upon the general subject of a mixed diet.

Some of the points made by Dr. Codman in his paper have raised a question in my mind. He speaks of certain animals presenting perfectly clean teeth, from the fact of their food being so simple that the tongue and the fluids of the mouth are able to cleanse the teeth. We find that condition in animals in their natural state. The teeth of the carniverous animals are as clean as the teeth of the herbiverous animals. They are as clean as the teeth of omniverous animals. All animals except the human eat their food uncooked; and I think the soft, glutinous matter of cooked food that sticks to the teeth has much to do with their decay and loss. If we take the human animals that present strong and clean teeth without the aid of tooth-brushes or powders, we find that they live very much in a state of nature; they are not congregated in cities or villages; they are not busied with intellectual pursuits, but they live an out-of-door life. The Rev. Mr. Murray, when he hailed from Boston, gave us some account of life in the Adirondacks, and he tells a story of a young man who was so debilitated when he went there that he could not get into that wild country without being carried, yet, after a residence of a few months there, he was able to endure the most fatiguing expeditions on foot without aid. When the young man who is debilitated by city life and heredity goes back to a more natural state of living, he finds that his body is built up, so that it can perform its natural functions. And it is because of these facts, which constantly come to our knowledge that I am a great skeptic in regard to the doctrine that our food has anything to do with the building up of our spiritual nature. We know that all parts of the body, the brain as well as the bones and muscles, are built up by the food which we eat; but I doubt very much whether the spiritual nature of man is so built up or changed, except our habits of taking food become gross and animal. There is a tendency to the animal nature and to the spiritual nature; but those tendencies are governed more by our habits than by the nature of the food which we take.

*Dr. Sudduth*—I would like to say a few words in regard to the connection of food supply with the cleanliness of the teeth of animals. How about the teeth of dogs? They are generally clean. They have the same cooked food that their masters have, and yet there is no decay in their teeth. There is only one article left out of the dog's diet, and that is sugar, that we take ourselves.

The omission of sugar makes the only difference between the food of the dog and that of his master. Now, from sugar comes fermentation; and decay is without question the result of fermentation and the production of lactic acid. The difference in the condition of the teeth of man and the lower animals is largely due to that difference in their food.

*Dr. Watkins*—Dr. Sudduth has spoken of dogs' teeth being always clean. I have noticed that the dog which is made a pet of and kept in the house and fed from the master's table invariably has a bad breath, and I have frequently seen tartar on their teeth also.

*President Brown*—Last year I wrote on this same subject myself; and I expected Dr. Atkinson to pitch into me, but he did not come. Now he makes the point that we should look at our organization to see what kind of food we should eat. That is out of the clouds. The position that I took in my paper was that we should try to decide this question by looking back to the natural or original food of man, and not by what we see to-day. In the glacial period the food of man was changed. I think there is no doubt but that before that age it was a fruit diet. It was then changed to an almost carniverous diet; and as the earth has changed we have become omniverous. The question resolves itself whether we are living correctly as omniverous animals or whether we should be fruitiverous. Dr. Winder says graminiverous animals have several stomachs, and that therefore man, who has but one, cannot be considered a graminiverous animal. I have never heard anybody claim that we are graminiverous, but it is exclaimed that we are naturally fruitiverous. In comparison with other animals we of course come nearest to the ape; the anatomy of the two is almost exactly the same; and if we take that as an example, the argument is surely in the direction of the fruitiverous theory.

*Dr. Ottolengui*—Dr. Watkins is right in claiming that the dog loses his teeth just about the same as we do when fed upon the same food. I have in my house a pet dog that lost his six molar teeth from pyorrhœa at about two or four years of age. It seems to me that the men who make a business of breeding dogs have really advanced to a very high point of knowledge on the subject of breeding those animals, and the perfecting of their several qualifications. They can produce almost any kind of a dog they may want. In the first place they pay a great deal of attention to the matter of heredity.

The matter of food supply has also been carried to a fine point by the breeders of dogs. The dogs are fed upon only one kind of food, and that is dog cake. It is not at all unpalatable to man. It is baked very hard and the dog is made to eat it dry. It is made of ground bones and a soup made of beef meat, and, I think, corn meal. Dogs fed upon that after two months will show a very marked improvement in their coats. They are fed only once a day, and are then given all they want to eat. I know persons who follow the same habit of eating only once a day and taking food that requires considerable mastication.

Adjourned until 8 o'clock P.M.: Dr. Codman's paper to be taken up for discussion.

### THURSDAY EVENING SESSION, JULY 18, 1888.

The roll was called by the Secretary.

The discussion of Dr. Codman's paper was taken up.

*Dr. Stockton*—We know that a great many people, when they come to this country from foreign countries, have very good teeth, and that in a few years after their arrival their teeth melt away. Is that due to the influence of diet, or to the climate of this country? Can Dr. Codman answer that question?

We know that the teeth of children here require more care between the ages of 10 and 20 years than at any other period. We are also told that the teeth of people who come from other countries, especially the peasant class, do not decay after they have been here until between the ages of 20 and 25 years. What is the reason of that? I would like an answer to this question, also, if the doctor can give it.

*Dr. Codman*—I am not able to answer Dr. Stockton's question as to whether the diet of the New England people, which I claim has improved, has had its effect on the teeth; but I think we all agree that whatever produces better constitutional effects produces better teeth. And I think dentists are hardly judges on the question of whether teeth have improved or not, because we see the decayed teeth and not the good ones as a rule; the best teeth keep out of our offices generally, and we are not so good judges of that perhaps, as some outside individual might be. I cannot say that the teeth of the New England people have improved in the last sixty years. I do believe that the diet of the New England people has had its effect, and that the consumptive tendency has been checked. There are not so many consumptives as there were in the past. And that is as much as to say that the food they eat is better assimilated.

With regard to foreigners coming here with good teeth and having them decay soon after they get here, I think possibly there is an error there, and that when they come here they only think they have good teeth when perhaps they have not. I find servants oftentimes coming to me with good looking teeth, which are really decayed, and it is only a question of time when they will come into the general average. You know also that there is a very wide departure when they get here, from their European diet. Nearly all the Catholic population of Europe are limited in the days when they may partake of animal food; one day in every seven it is prohibited, and there are six weeks in Lent when they cannot take it. Further than that, there are those who cannot get animal food, neither can they get sugar, tea, coffee, etc. I have been told that in some parts or Germany sugar is so rare an article among the common people, that when they get a piece of it they carry it upon a string and each one of the family takes a lap at it. Here instead of considering it a luxury seldom tasted, they go into a barrel of sugar with savage appetites, and they eat it plentifully as well as other things which they were not accustomed to in their own country, and that unaccustomed food produces a disturbance of the system. Even the simplest things taken by a person that is not accustomed to them will often cause disturbance to the system; and whatever disturbs the normal condition of the system has its effect upon the tissues.

Something has been said about dogs, and the question was answered pretty fairly, but I have a patient who says that when his dogs are in the country they never have any tartar on their teeth, but when they come to town and live again on the food from the family table their teeth become covered with tartar. The reason of that is that in town they have no bones to knaw while in the country they scratch and root around and keep their teeth clean.

We must rid ourselves of the prejudice which is abroad among scientific men in regard to the difference in foods. There is not a great deal of difference between the mutton which you eat and the vegetable food which the sheep eat. The difference is mainly that the sheep ate the vegetable food first.

It was not many years ago when it was thought to be almost impossible for a person to live without animal food; the man was thought to be crazy who spoke of such a thing; but as our observation increases our ideas advance, we know more of the habits of other nations, and we find that some nations live almost entirely upon vegetables and fruits. We also know that some of the strongest people who have lived in the world, and who live on the Mediterranean, would take a package of three to six hundred pounds and trot off with it on their backs over the country, and they eat very little or no meat. The Japanese boys will take a lady in a chair and trot away at the rate of six or seven miles an hour, and they live almost entirely upon fruits and vegetables. But my idea is not, as some gentlemen have suggested, that our normal diet is grain, but the man is intended to be fruitiverous. Nature hands your fruit to you in the most beautiful shapes, such as the peach, and the plum, and the walnut, which are held out to you by the tree, and they are very tempting and very wholesome foods. I thank you for your kind reception of the paper, and I am pleased to be with you on this occasion.

Subject passed.

EVENING SESSION.

Dr. Sudduth showed with the magic lantern a large number of photo-micrographs of sections of the tissues of the lower animals in various stages of embryonic life. He prefaced his remarks with a graphic description of his idea of the beginning of life on the face of the earth.

After describing the pictures shown, Dr. Sudduth said: "In showing you these various stages of development of life from a microscopic point of view, I have tried to bring out the idea of the separate entity in cell life; the history of the development of the individual is simply that of the development of the individual cell. The ova is nothing more than a separate cell that has the power of reproducing itself. It exemplifies the division of life in the cell. All the changes of the body spring from these separate entities that we find here represented.

"All change must begin in these ultimate principles. We call that the cell an ultimate principle because we do not know any other. These cells spring from pre-existing cells. The *de novo* origin of life is impossible. The only way in which life can be developed is from pre-existing life. I want to call attention to the fact that the green plant was the first point in the development of life. I believe the green plant was produced by creative fiat. The development of the green plant *de novo* is also an impossibility. One life descends and blends into the other life. Man is a creature of heredity and environment, subject to the changes and vicissitudes of life, and his nature is changed and modified by the circumstances under which he exists.

The question of diet was brought up to-day. We are creatures of environment, but we have the power of modifying our environment to a greater or less extent. That man is changing all the time cannot be questioned. We have evidence of that as far as we can go back in history. We adapt ourselves to different circumstances, and there has been no study in the world's history that promises more at the present day than that of personal and corporate hygiene. If we can better our environment, we better the conditions of life, and thereby lengthen life. Physiological chemistry is taking the lead to-day, and the man who can take up that study and follow it out will give us the most light in the future. There has never been a time in the world's history when we have had so much promise of benefit to mankind as at present, and it is largely due to our increasing knowledge of what true hygienic conditions are.

*Dr. Atkinson*—We have seen a very beautiful representation of the development of tissues, and have been told that there must be a cell for these changes to take place. Now there is a correlative of that, that if the cell grows from its nucleus it must naturally have increment of substance to increase the size of that nucleus, and to make the division that has been portrayed as actually occurring in the rabbit's egg, and in the unimpregnated chicken's egg. How came about the possibility of providing and elaborating the new impulse to that egg, that had to be fed to a certain point of ripeness to make it possible to establish a line of sympathy between the individuals, so that when thrown together in an undifferentiated mass, they become organized into semens, the combination of which produces the veritable germ.

We must understand that fine feeding, or else we are simply beating the air and talking about beautiful things without really following the line of evolution of the radiancy that is stored in these little bodies that constitute the papulum, the differentiations of which give us the classification of creatures. Hæckel said that the protista was a kingdom between the vegetable and the animal kingdoms.

We must be in our intellects and affections on a plane of fraternity and love of the truth, and until we do we will not make the progress that we should, even after we have had such a display as we have had to-night.

They tell us of chaos. We can never travel towards the real foundation until we comprehend chaos. Chaos is the conglomerated mass of planetary substance that is capable of being developed into planets and planetarian inhabitants, the discriminations and divisions of which constitute the classifications in natural history. We want to give a specific alphabetical statement to students who attempt to investigate these things. First chaos, next protoplasm, next protista, then the mineral kingdom, the typical form of which is the crystal, next the vegetable kingdom, characterized by the cell, as the doctor called it. The function of the cell is to vegetate. Then we come to the animal kingdom. What is the principal example of that? It is the corpuscle. It is not a cell. It is a cell and something more; just as the first protista was chaos and something more; the stored radiancy embodied in the planetary substance out of which all the body of the planet and its inhabitants take their origin.

*Dr. Sudduth*—You said the corpuscle was more than the cell. I want to get your idea of what you term a cell.

*Dr. Atkinson*—The cell is the primal element of vegetable activity. It is simply a lump of jelly that is non-nucleated. I have written upon this subject, and I am pained to say that not a single man has manifested the disposition to read understandingly what I have said. Some learned men have said I spoke ambiguously.

*Dr. Thayer*—What is a corpuscle?

*Dr. Atkinson*—A corpuscle is a little body.

*Dr. Thayer*—How does it differ from the cell?

*Dr. Atkinson*—It differs in its promise as a body.

*Dr. Ottolengui*—The paper teaches us that these cells are individuals. Dr. Heitzmann teaches that they are nothing of the kind.

*Dr. Atkinson*—It takes two lies to make a truth apparent. They are both, and they are neither. That was undoubtedly at one time a globular body (referring to the screen). That is developed here first. It is globular; then they run together and become columnar; then they press against each other on all sides and flatten and become cuboidal. Then all outside of that (referring to the screen) is squamus or pavement epithelium. As they approach the surface, the flattening represented here becomes more marked, and they are ultimately shed as dry scales with a central nucleus. That may be round, cylindrical, cuboidal, or squamus, according to its age and mission it is fulfiling. They have muddled the truth about that, but I do not think he (Sudduth) did. Take that and transplant it anywhere where the epithelial body or malpegian layer has been taken off, and you have enough left of the capillary layer and the true skin, cutis vera, and it is capable of feeding and will make a beautiful epithelium and a new surface to the skin. The dandruff that we see on persons who are poorly groomed is shed epitheliel scales. We should understand how the feeding process goes on, and how in the process of digestion something is laid by for future use, as we bank some of our money; and if we would observe the process of respiration we would have a key to unlock this mystery. We get an oxide when not breathing. When we inspire and bring the atmosphere into the air vesicles a transition takes place. And what does that do? Just 4 per cent. of the oxygen that has been grasped by the lungs is appropriated, 96 per cent. is killed, is not fit to be used until it has passed the vegetable kingdom to be rejuvenated by discharging its carbon and taking in oxygen. The next step is to get rid of the breath which is expired. If we were learners and would learn the lesson that this example teaches us, it would give us a key to unlock every one of the modes of nutrient motion from the crystal to the cell and the corpuscle, the tissue, organ, system and consciousness. But I have expressed the entire roll from A to izzard.

*Dr. Ottolengui*—I do not understand your answer.

*Dr. Atkinson*—What is our body? Nothing but a single substance, an amœba, a mere big lump of jelly that differentiates into various tissues and organs. It is proved by many examples in the practice of medicine, in physiology, and the researches of the micologist and naturalist. The alimentary, respiratory, and urinary tracts are plainly laid out in the human amœba as foresteps to the perfect organs of the body; they are made up of indifferent corpuscles that are nothing but lumps of jelly. Then heredity is involved, and under favorable circumstances there is produced a similar ova to that from which we are told that body was developed. But where did the first one come from if this is true?

*Dr. Sudduth*—It was created by the power behind us.

*Dr. Atkinson*—He uses the word creation. The idea is right. "In the beginning God made man in his own image; in the image of God made he him; male and female created he them." There is first a single cell, holding the potentiality of father and mother. First is the maternal or love side; then the father, or wisdom; and then in the ongoing of these two, love and wisdom, the divine logos gives form and conformation to all planets and planetary inhabitants.

The potentiality that resides in the human amœba, that is the ovum already vivified, lays the foundation of the three embryonal sheets, so-called, the epiblast, hypoblast and mesoblest, the enfoldings of which give us the entire system of primal parts. Every time that you have a reproduction of tissue it has to go through that same process that I speak of. First, indiscriminate chaos; then completely digested food or peptones; then protoplasmic mass; then the embryonal corpuscle, out of which all the tissues arise, as exemplified in all reproduction of structure where there is fracture of the tissues; if they are favorably situated, they repeat the embryonal condition and series of changes, so that they are undistinguishable from the original material, and are not distinguishable as scar tissue; but where the circumstances are minus in organizing power, the white connective tissue corpuscles prevail to such an extent as to make the dense scar tissue that is known to prevail so extensively in burns and other mutilations, and which is less endowed with organizing power, and most liable to retrograde when under debilitating conditions of constitutional origin.

*Dr. Sudduth*—What are corpuscles, and what differentiates them? Are we able to see a corpuscle?

*Dr. Atkinson*—Yes. These (referring to screen) are corpuscles, and may be the beginning of what you call cell division in proliferation of the new structure. You said they were nucleated cells. These little spots are neuclei. They are being merged into one another; so when we lift the connective tissue or epithelium from the papillæ of the true skin, we may have normal reproduction of the structure, or that which is less endowed and called scar tissue, or simply white connective tissue corpuscles binding the fractured parts together. These are all limitary tissues. When they are destroyed you get union between the horny layer, which is the true protection against infection.

They do not know what Heitzmann meant by saying that all protoplasm is organized. He has shown that in the red and white human corpuscle, in which the reticulum of what he calls living matter embraces the fluid or non-living portion of the corpuscle. Our American investigators have looked and looked and still they look to the east, to Europe, for the interpretation of these fine questions, but you have followed the line of investigation and treatment of the disease of Frederick III under Mackenzie, and the German physicians have seen that no single one of them made any specific reply to the question of whether the formation was or was not malignant; but they hedged and quarreled among themselves, and Mackenzie stands the only clean-cut man in the lot. He said in the start that he did not know whether it was malignant or not. They cut some tissue and sent it to Virchow, and he said he could not find anything in it that would warrant him in saying it was malignant. After the patient died then they decided that it was malignant cancer. They give us the old saw respecting carcinoma; if the patient dies, then it was cancer; if he don't die then it was not cancer. So you never cure cancer. There is no microscopical demonstration that shall teach us whether a tumor is malignant or not. It is not possible at this day to determine whether a tissue is malignant or non-malignant by microscopic means; and I do not hope to see it until we get deeper into physiological chemistry and understand what is the constitution of a chemical individual, what is aptomaine, a poison, or a leucomaine, and know more of the finer foods, to the tissues than is known to physiologists.

*Dr. James Truman*—Dr. Sudduth stated that there is no new life, that all cells come from cells, and all life comes from life, and he spoke several times of creation as though at some period there was no cell; that it was created. Now if all cells came from cells, and all life comes from preceding life, then there must be an eternity of life, a continuous circle, and there is no necessity of creation at all. I don't understand his idea of creation under those circumstances. It seems to me unscientific,—unless there has been a beginning there never can be an end.

*Dr. Sudduth*—This question is a very hard one to answer. It is a question that is outside of demonstration. I have tried to-night to confine myself to that which can be demonstrated. The beginning of life can only be reasoned out. We know that we have passed through a stage of evolution. Spontaneous generation was called out and followed up, bnt did not go far until it was proven to be a chimera. We find that life is persistent throughout the atmosphere. There may have been a point, as evolutionists claim, in the existence of the earth when the circumstances were just right, and when spontaneous generation of life was possible; but all our analogies teach us the opposite. So we come back to that creative fiat that I acknowledge, and while the eye cannot penetrate the vale it recognizes a creator behind. This life is not eternal. Dr. Atkinson has misunderstood me. I have argued that behind all this there is something that we cannot fathom, which directs and guides this form of life. I think physical life is limited.

*Dr. Ottolengui*—I understand that Dr. Sudduth claims that these cells are individuals which can reproduce themselves, just the same as a plant which throws off a seed from which comes another plant entirely separate and distinct, except in the matter of heredity. Dr. Heitzmann teaches the opposite. He says that those cells, instead of dividing into two separate parts, simply grow out until the middle of each collapses, and by the time it has collapsed the two bulging sides are as large as the original cell was, and that process goes on and on, yet they are the same original cells. I want to know exactly what evidence he has that Dr. Heitzmann is wrong and that he is right in the matter.

*Dr. Sudduth*—We have here a display of the Malpegian layer of the skin, that makes the lining membrane of the mouth. Here is represented the epiblast, the outer of the three layers, and this represents the mesoblast, or middle layer. This Malpegian layer is made up of small granular bodies, which are called nuclei. They are the infant cells. Within each are seen numerous dark granular particles, which we call nucleoli. All cellular change begins within the nucleus by a re-arrangement of the nucleoli, by a process termed karyokinsis. The nucleus then divides; after which the cell body divides. The subject was passed.

[To be Continued.]

